# A Genome-Wide Association Study to Identify Diagnostic Markers for Human Pathogenic *Campylobacter jejuni* Strains

**DOI:** 10.3389/fmicb.2017.01224

**Published:** 2017-06-30

**Authors:** Cody J. Buchanan, Andrew L. Webb, Steven K. Mutschall, Peter Kruczkiewicz, Dillon O. R. Barker, Benjamin M. Hetman, Victor P. J. Gannon, D. Wade Abbott, James E. Thomas, G. Douglas Inglis, Eduardo N. Taboada

**Affiliations:** ^1^National Microbiology Laboratory at Lethbridge, Public Health Agency of Canada, LethbridgeAB, Canada; ^2^Department of Biological Sciences, University of Lethbridge, LethbridgeAB, Canada; ^3^Lethbridge Research and Development Centre, Agriculture and Agri-Food Canada, LethbridgeAB, Canada

**Keywords:** *Campylobacter jejuni*, genome sequence, genome-wide association study, clinical association, molecular marker discovery, linkage analysis, molecular risk assessment

## Abstract

*Campylobacter jejuni* is a leading human enteric pathogen worldwide and despite an improved understanding of its biology, ecology, and epidemiology, limited tools exist for identifying strains that are likely to cause disease. In the current study, we used subtyping data in a database representing over 24,000 isolates collected through various surveillance projects in Canada to identify 166 representative genomes from prevalent *C. jejuni* subtypes for whole genome sequencing. The sequence data was used in a genome-wide association study (GWAS) aimed at identifying accessory gene markers associated with clinically related *C. jejuni* subtypes. Prospective markers (*n* = 28) were then validated against a large number (*n* = 3,902) of clinically associated and non-clinically associated genomes from a variety of sources. A total of 25 genes, including six sets of genetically linked genes, were identified as robust putative diagnostic markers for clinically related *C. jejuni* subtypes. Although some of the genes identified in this study have been previously shown to play a role in important processes such as iron acquisition and vitamin B_5_ biosynthesis, others have unknown function or are unique to the current study and warrant further investigation. As few as four of these markers could be used in combination to detect up to 90% of clinically associated isolates in the validation dataset, and such markers could form the basis for a screening assay to rapidly identify strains that pose an increased risk to public health. The results of the current study are consistent with the notion that specific groups of *C. jejuni* strains of interest are defined by the presence of specific accessory genes.

## Introduction

*Campylobacter jejuni* is one of the leading causes of bacterial foodborne gastroenteritis in the world; it is estimated to be responsible for as much as 14% of all cases of diarrheal disease, translating to more than 400 million cases of campylobacteriosis annually (Duong and Konkel, 2009). In Canada, annual incidence rates nearing 30 cases per 100,000 individuals have been reported (Galanis, 2007), although statistical models that account for unreported and undiagnosed cases suggest this rate could be as high as 447 cases per 100,000 individuals (Thomas et al., 2013). While a majority of cases are self-limiting, post-infection complications, such as Guillain-Barré syndrome can be life threatening (Nachamkin et al., 1998; Nachamkin, 2002). *Campylobacter jejuni* is commonly isolated from the gastrointestinal tract of many different wild and domesticated species, including companion animals and food animals such as poultry and cattle (Lastovica et al., 2014). Faecal contamination from carrier animals is considered to be a primary source of *C. jejuni* in the environment and on food products (Koenraad et al., 1997). This bacterium is highly prevalent in raw poultry meat and poultry by-products (Suzuki and Yamamoto, 2009; Williams and Oyarzabal, 2012), and the consumption and handling of contaminated poultry products is thought to be the primary source of exposure leading to human infection. Nonetheless, the epidemiology of campylobacteriosis is complex, with a large number of cases that appear to be sporadic (Silva et al., 2011), a range of animal and environmental reservoirs (Whiley et al., 2013), and multiple potential routes for the introduction of *C. jejuni* into the food chain as well as non-food-related pathways of exposure (Pintar et al., 2016).

Although epidemiological evidence suggests that not all *C. jejuni* strains or genetic lineages pose an equal risk to human health, our current understanding of *C. jejuni* subtype-dependent pathogenesis is incomplete. In contrast to other enteric pathogens, *C. jejuni* does not possess a number of the classical virulence factors (e.g., Type III or Type IV secretion systems, enterotoxins) found in other pathogens (Havelaar et al., 2009). Previous studies have identified genetic determinants that are important for *C. jejuni* pathogenicity (Dasti et al., 2010), but they are generally conserved across the species. Therefore, these factors have little predictive power for the identification of isolates with a higher propensity to cause disease in humans.

With the advent of inexpensive and high-throughput whole genome sequencing, Genome Wide Association Studies (GWAS) are increasingly being applied to bacterial genomics as tools for the identification of genetic markers associated with a phenotype or trait of interest (Read and Massey, 2014). GWAS represent a “top-down” approach to molecular marker discovery because the genomic content of “test” and “control” groups is compared and analyzed to identify genetic variation that is strongly associated with a given trait. This is in contrast to “bottom-up” approaches where individual genetic factors are manipulated to observe a phenotypic effect. The utility of GWAS lies in their ability to test many genetic factors in order to reveal associations with the phenotype of interest without *a priori* assumptions on the specific biological processes involved (Read and Massey, 2014). GWAS have been utilized to identify mutations and other polymorphisms associated with antibiotic resistance in *Mycobacterium tuberculosis* (Farhat et al., 2013), *Staphylococcus aureus* (Alam et al., 2014), and *Streptococcus pneumoniae* (Chewapreecha et al., 2014). In *Campylobacter*, GWAS have been used to identify genetic factors related to the Guillain-Barré Syndrome (Taboada et al., 2007), host adaptation in *C. jejuni* and *Campylobacter coli* (Sheppard et al., 2013), and has recently been used to identify markers associated with the survival of *C. jejuni* in the poultry production chain (Yahara et al., 2016).

In this study, we have used isolates from the Canadian *Campylobacter* Comparative Genomic Fingerprinting Database (C3GFdb) to perform a GWAS aimed at identifying genetic determinants preferentially found among *C. jejuni* lineages associated with human disease. Comparative Genomic Fingerprinting (CGF) (Clark et al., 2012; Taboada et al., 2012) has been used as the primary tool for subtyping of *C. jejuni* isolates made available through a range of projects in Canada, including the FoodNet Canada sentinel surveillance program, the Canadian Integrated Program for Antimicrobial Surveillance, the Canadian Food Inspection Agency’s microbiological baseline survey of poultry, and several projects that incorporate human, food animal, wild animal, retail food, and environmental sampling activities. The C3GFdb currently contains subtyping data for 24,142 *Campylobacter* isolates from human (*n* = 4,697), animal (*n* = 14,750), and environmental (*n* = 4,457) sources from across Canada, representing 4,882 unique subtypes. It also contains basic epidemiological metadata for each isolate including host source, date and location, which facilitates contextualization of subtypes within the broader population structure of *C. jejuni* circulating in Canada.

The goal of the current study was to identify accessory genes with a statistically significant difference in carriage rates in two *C. jejuni* cohorts that differ in terms of their association with human campylobacteriosis. These genes could be used as diagnostic markers for molecular-based risk assessment and the rapid detection of *C. jejuni* isolates that pose the greatest risk to human health.

## Materials and Methods

### Strain Selection

**FIGURE 1:**
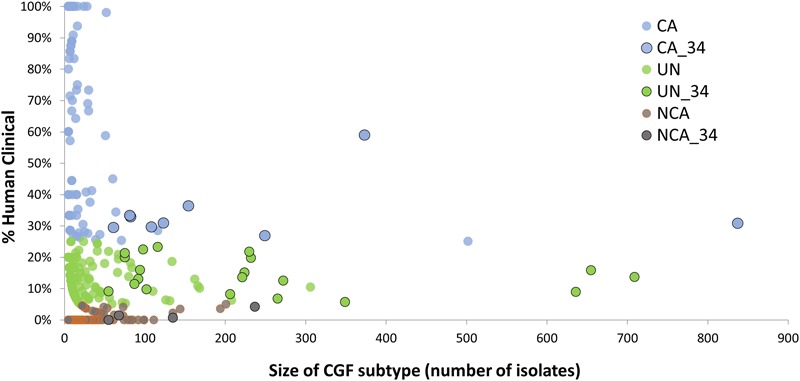
Identification of CGF subtypes for GWAS analysis of Clinically-Associated (CA) vs. Non-Clinically-Associated (NCA) *Campylobacter jejuni* subtypes. The C3GFdb was used to identify 166 *C. jejuni* isolates for whole genome sequencing from 34 highly prevalent CGF subtypes (black outline) that together account for nearly 31% of all isolates in the database, and over 55% of all isolates from the 100 most prevalent CGF subtypes circulating in Canada. These subtypes exhibit differences in their association with human campylobacteriosis, and sequence data from representative isolates was used in a genome-wide association analysis aimed at identifying accessory genes associated with clinically relevant *C. jejuni* subtypes.

A total of 166 *C. jejuni* isolates representing 34 of the 100 most prevalent CGF subtypes circulating in Canada were selected from the C3GFdb for whole genome sequencing (Supplementary Table S1). The selected isolates and their respective subtypes represented approximately 31% (7,407/24,142) of all isolates in the database and over 55% (7,407/13,367) of the isolates from the 100 most prevalent CGF subtypes (**Figure 1**). They have been observed in multiple provinces, sources and hosts, and over multiple years, suggesting that they are endemic and in wide circulation. The dataset selected for WGS was comprised of 72 isolates from animals or retail meat, 54 isolates from environmental sources, and 40 isolates from human clinical cases (**Table 1**).

**Table 1:** Epidemiological characteristics of 34 CGF subtypes targeted for whole-genome sequencingbased on the Canadian *Campylobacter* Comparative Genomic Fingerprinting Database (C3GFdb).

				Proportion of isolates in subtype (%)^4^			
CGF Subtype	Cohort^1^	Cluster Size^2^	Cluster Rank^3^	H	A	E	U
0169.001.002	CA	837	1	30.8%	62.7%	6.3%	0.1%
0695.006.001	UN	709	2	13.7%	80.4%	5.9%	0.0%
0083.001.002	UN	655	3	15.9%	83.2%	0.8%	0.2%
0926.002.001	UN	636	4	9.0%	74.2%	16.8%	0.0%
0044.003.001	CA	373	6	59.0%	40.5%	0.5%	0.0%
0957.001.001	UN	349	7	5.7%	69.6%	24.6%	0.0%
0853.011.001	UN	272	9	12.5%	87.1%	0.4%	0.0%
0882.005.001	UN	265	10	6.8%	81.1%	9.4%	2.6%
0982.001.002	CA	249	11	26.9%	68.3%	4.8%	0.0%
0811.009.002	NCA	237	12	4.2%	43.9%	51.9%	0.0%
0735.005.001	UN	232	13	19.8%	66.8%	13.4%	0.0%
0253.004.001	UN	230	14	21.7%	75.2%	3.0%	0.0%
0960.007.001	UN	224	15	15.2%	76.8%	5.4%	2.7%
0731.001.005	UN	221	16	13.6%	81.9%	4.5%	0.0%
0923.002.001	UN	206	18	8.3%	61.7%	30.1%	0.0%
0269.004.001	CA	154	24	36.4%	63.6%	0.0%	0.0%
0811.008.001	NCA	135	26.5	0.7%	45.9%	53.3%	0.0%
0173.004.001	CA	123	31	30.9%	57.7%	11.4%	0.0%
0173.002.004	UN	116	32.5	23.3%	76.7%	0.0%	0.0%
0933.004.002	CA	108	36	29.6%	65.7%	4.6%	0.0%
0893.001.001	UN	102	37	9.8%	82.4%	7.8%	0.0%
0933.008.001	UN	98	40	22.4%	75.5%	2.0%	0.0%
0949.001.002	UN	94	41	16.0%	72.3%	11.7%	0.0%
0960.003.002	UN	92	42.5	13.0%	67.4%	19.6%	0.0%
0904.002.002	UN	87	44	11.5%	74.7%	12.6%	1.1%
0103.001.002	CA	82	48.5	32.9%	67.1%	0.0%	0.0%
0077.001.003	CA	81	51	33.3%	66.7%	0.0%	0.0%
0238.007.002	UN	75	55.5	20.0%	80.0%	0.0%	0.0%
0260.007.001	UN	75	55.5	21.3%	78.7%	0.0%	0.0%
0844.001.001	NCA	68	63	1.5%	23.5%	75.0%	0.0%
0253.001.002	CA	61	69.5	29.5%	68.9%	0.0%	1.6%
0535.001.003	UN	55	76.5	9.1%	36.4%	54.5%	0.0%
0817.003.001	NCA	55	76.5	0.0%	20.0%	80.0%	0.0%
0083.007.001	CA	51	81	58.8%	41.2%	0.0%	0.0%

*^1^Cohorts: Clinically Associated (CA); Non-Clinically Associated (NCA); Undefined (UN). ^2^Number of isolates observed with the CGF subtype in the C3GFdb. ^3^Rank of CGF subtype (based cluster size) in the C3GFdb. ^4^Proportion of isolates in the subtype from Human (H), Animal (A), Environmental (E), and Unknown (U) sources.*

### Genome Sequencing, Assembly, and Annotation

Sequencing was conducted at Canada’s Michael Smith Genome Sciences Centre, BC, Canada using the Illumina HiSeq 2000 platform. Whole genome sequence data for this study has been deposited in the Sequence Read Archive (SRA) at the National Center for Biotechnology Information (NCBI) under the BioProject PRJNA368735. Draft *de novo* genome assembly of paired-end reads was performed using SPAdes v.2.4.0 (Bankevich et al., 2012) with pre-assembly BayesHammer read correction, default k-mer size testing options, and post-assembly Burrows Wheeler Aligner mismatch correction. Contigs with low coverage or shorter than 500 bp were removed from all subsequent analyses. Genome assembly quality was assessed using QUAST v.2.1 (Gurevich et al., 2013). Prediction of Open Reading Frames (ORFs) and annotation was performed using the PROKKA pipeline v.1.5.2 (Seemann, 2014) using a custom database of non-redundant gene sequences representing five complete and well-annotated *C. jejuni* reference genomes available from NCBI (Supplementary Table S2).

### Definition of a *C. jejuni* Reference Pan-Genome for the Dataset

Predicted ORFs were queried using a reciprocal best hit approach (Moreno-Hagelsieb and Latimer, 2008; Ward and Moreno-Hagelsieb, 2014) with BLAST v 2.2.29 (Camacho et al., 2009) in order to define a reference pan-genome, the non-redundant set of genes for a set of genome sequences (Méric et al., 2014). Paired BLAST queries were treated as *orthologous* if they shared ≥80% sequence identity and ≥50% alignment coverage and a single exemplar was included in the pan-genome. The pan-genome defined using this process was used in the subsequent GWAS.

### Genome Wide Association Study

Carriage across the dataset of all genes representing the pan-genome was assessed by BLAST analysis. The nucleotide sequence of each gene was queried against the 166 draft genome assemblies using Blastn. Genes were considered to be *present* if a hit representing ≥80% sequence identity over ≥50% of the length of the query gene was found and considered *absent* otherwise. In order to facilitate statistical comparison, subtypes were defined as either Non-Clinically Associated (NCA; ≤5% human clinical isolates), Undefined (UN; 5–25% human clinical isolates), or Clinically Associated (CA; ≥25% human clinical isolates). The statistical significance of each gene (*p* < 0.05) was defined based on its carriage rate in the CA and NCA cohorts and was computed using Fisher’s Exact test statistic in GenomeFisher^1^; *p*-values were adjusted for multiple testing using the method of Holm (Holm, 1979; Aickin and Gensler, 1996). Statistically significant genes were subjected to further analysis and validation as outlined below.

#### *In Silico* Validation of Putative Diagnostic Marker Genes

In order to select markers with the highest potential for downstream assay development, candidate genes identified by the GWAS analysis were filtered in a stepwise process according to the following conditions: (1) complete absence in the NCA cohort and presence in ≥50% of CA genomes; (2) high sequence identity (>99%) and complete, or near complete, conservation of sequence length (>90%) in the corresponding orthologous gene, when present, among a set of reference genomes (**Table 2**); and (3) statistical significance (*p* < 0.05) when the NCA cohort was compared to a combined CA+UN cohort, in which the UN (i.e., undefined) genomes were treated as CA and pooled with the CA genomes. Genes that passed all criteria were selected for *in silico* validation using a larger set of genome sequences. This validation dataset was created by combining genomes sequenced in house as part of current or previous studies (*n* = 325) and additional genomes acquired from public repositories (*n* = 3,955). Publicly available genomes were restricted to those with available epidemiological data (e.g., sample source, country of origin, etc.). To facilitate assignment into NCA, UN, and CA cohorts, *in silico* CGF was performed on these genomes using MIST (Kruczkiewicz et al., 2013), with a concordance between CGF profiles predicted *in silico* and those generated in the laboratory estimated to be 96.8% on a subset of 325 isolates (12,583/13,000 matching loci; data not shown); only genomes from CGF subtypes previously observed in the C3GFdb were retained in the validation set (*n* = 3,902). Each genome was designated to its respective cohort based on the corresponding epidemiological data of the *in silico* CGF subtype. Finally, the putative diagnostic genes identified by the GWAS using the original set of 166 genomes were tested for statistical significance with the expanded cohorts. The combinatorial effect of different subsets of markers was also assessed to determine if a reduced number of markers could be applied to detect clinically related *C. jejuni* subtypes without a subsequent loss of sensitivity.

**Table 2:** Significant genes observed after GWAS analysis of genome sequences from representative Clinically Associated (CA) and Non-Clinically Associated (NCA) *C. jejuni* subtypes.

	*p*-value^1^					
Marker	Raw	Holm-corrected^2^	11168 Ortholog	Gene name	Function	Linkage group
11168_00051	4.29E-10	8.39E-07	*Cj0055c*		Hypothetical protein	LG1
11168_00052	5.28E-10	1.03E-06	*Cj0056c*		Hypothetical protein	
11168_00169	3.36E-11	6.61E-08	*Cj0177*		Putative iron transport protein	LG2
11168_00170	3.36E-11	6.61E-08	*Cj0178*		Putative TonB-denpendent outer membrane receptor	
11168_00171	3.36E-11	6.60E-08	*Cj0179*	*exbB1*	Biopolymer transport protein	
11168_00172	3.36E-11	6.60E-08	*Cj0180*	*exbD1*	Biopolymer transport protein	
11168_00173	3.36E-11	6.60E-08	*Cj0181*	*tonB1*	TonB transport protein	
11168_00230	6.12E-19	1.21E-15	*Cj0246c*		Putative MCP-domain signal transduction protein	
11168_00243	6.48E-34	1.28E-30	*Cj0259*	*pyrC*	Putative dihydroorotase	LG3
11168_00244	3.10E-27	6.14E-24	*Cj0260c*		Small hydrophobic protein	
11168_00248	6.57E-25	1.30E-21	*Cj0264c*		Putative molybdopterin containing oxidoreductase	LG4
11168_00249	6.57E-25	1.30E-21	*Cj0265c*		Putative cytochrome C-type haem-binding Periplasmic protein	
11168_00277	1.30E-17	2.57E-14	*Cj0295*		Putative acetyltransferease	LG5
11168_00278	1.35E-18	2.66E-15	*Cj0296c*	*panD*	Aspartate 1-decarboxylase precursor	
11168_00279	1.35E-18	2.66E-15	*Cj0297c*	*panC*	Pantoate-beta-alanine ligase	
11168_00280	1.35E-18	2.66E-15	*Cj0298c*	*panB*	3-methyl-2-oxobutanoate hydroxymethyltransferase	
11168_00281	1.09E-16	2.15E-13	*Cj0299*		Putative periplasmic beta-lactamase	
11168_00703	6.98E-24	1.38E-20	*Cj0731*		Putative ABC transport system permease	
11168_00718	3.36E-11	6.59E-08	*Cj0753c*	*tonB3*	TonB transport protein	LG6
11168_00719	3.36E-11	6.59E-08	*Cj0755*	*cfrA*	Ferric enterobactin uptake receptor	
11168_01072	4.90E-11	9.59E-08	*Cj1122c*		Putative integral membrane protein.	
11168_01201	6.12E-19	1.21E-15	*Cj1255*		Putative isomerase	
11168_01309	5.30E-15	1.04E-11	*Cj1365c*		Putative secreted serine protease	
11168_01519	4.29E-10	8.39E-07	*Cj1585c*		Putative oxidoreductase	
11168_01610	4.29E-10	8.38E-07	*Cj1679*		Hypothetical protein	
06_2866_00597	6.89E-28	1.36E-24			Di-/tripeptide transporter	
06_7515_00723	4.19E-16	8.24E-13			Prophage Lp2 protein 6	
07_0675_00227	2.62E-11	5.15E-08		*tetO*	Elongation factor G	

*^1^*p*-value based on 2-tailed Fisher’s Exact Test. ^2^*p*-values were adjusted using the Holm-correction (Holm, 1979).*

## Results and Discussion

### Genome Sequencing, Assembly, and Annotation

The quality of the *de novo* assembly of the 166 genomes selected as representatives of 34 highly prevalent CGF subtypes in Canada was assessed using QUAST (Gurevich et al., 2013). The average number of reads produced for each genome was 4,161,271 (±1,223,304), for an average coverage of 253× (±74.7×). Individual genome assemblies had an average of 67 (±27) contigs and an N75 of 34,631 bp (±13,815 bp). All genome assemblies had additional parameters in range with what has typically been observed for *C. jejuni.* The average assembly length (1,660,986 ± 51,283.5 bp), predicted ORFs (1,719 ± 71), and %G+C (30.4 ± 0.13%) were typical of *C. jejuni* genome assemblies available in the public domain. Annotation of the 166 draft genomes from this study using the PROKKA pipeline (Seemann, 2014) resulted in the identification of 291,502 ORFs. The genome of strain NCTC 11168, which has been completely sequenced (Parkhill et al., 2000), was included in the analysis as a control to assess the completeness of the ORF prediction and annotation process. The original annotation of NCTC 11168 predicted 1,654 ORFs, while a subsequent re-annotation predicted 1,643 ORFs (Gundogdu et al., 2007); in our analysis, the PROKKA pipeline predicted 1,659 ORFs. This small discrepancy is related to the advanced curation used in the re-annotation of NCTC 11168, which resulted in the merging and removal of coding sequences belonging to pseudogenes and phase variable genes. The pan-genome established using this dataset consisted of 3,358 unique ORFs, of which 1,377 were present in all genomes (i.e., core genes) and 1,981 were present in a varying number of genomes (i.e., accessory genes).

### Genome Wide Association Study

Of the 166 C. *jejuni* isolates selected for this study, 35 were assigned to the NCA cohort and represented four different CGF subtypes, 80 were assigned to the UN cohort and represented 20 CGF subtypes, and 51 were assigned to the CA cohort and represented ten CGF subtypes (**Table 1**). A GWAS was performed in order to identify accessory genes with a biased distribution in CA and NCA cohorts. Although in principle GWAS can be used to identify genetic variation ranging from SNPs to indels involving multiple genes, we chose to focus on accessory genes, as they have excellent potential for the development of rapid, robust, and inexpensive PCR-based diagnostic assays for screening of large numbers of strains. At the same time, it is important to note that other forms of genetic variation may represent valuable targets for tracking strains of interest. Recently, Clark et al. (Clark et al., 2016) showed that large-scale chromosomal inversion could be used to distinguish a subset of outbreak-associated isolates from epidemiologically unrelated co-circulating isolates.

**FIGURE 2:**
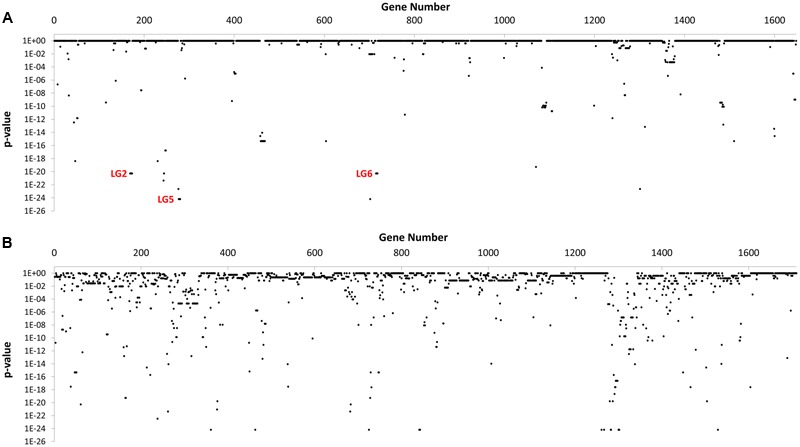
GWAS-based Identification of genes with significant differences in carriage between NCA and CA cohorts. The distribution of *p*-values observed for 3,358 genes in the *C. jejuni* pangenome computed for this study after Genome Fisher analysis of gene carriage data for CA and NCA cohorts. **(A)** Genes from NCTC 11168 genome strains (*n* = 1,648). **(B)** Genes from all other genomes (*n* = 1,710).

In total, 595 genes showed statistically significant differences in carriage between NCA and CA cohorts (*p* ≤ 0.05) (**Figure 2**). Of these, 71 genes were completely absent from the NCA cohort but were present in at least ≥50% of isolates in the CA cohort (Condition 1), and 63 of these genes also maintained high sequence identity (>99%) and near complete sequence coverage (>90%) compared to their respective reference genes (Condition 2). Of these, 28 continued to exhibit robust statistical significance when the NCA cohort was compared to a pooled cohort comprised of all UN and CA genomes (Condition 3). These include six sets of genes that appear to be found in linkage groups (**Table 2**), with members of each linkage group possessing similar rates of carriage in the dataset. Since linked genes, which are located adjacently on the chromosome, tend to be functionally related and are typically transmitted as a functional unit (Muley and Acharya, 2013), it is likely that their identification in this study was not due to spurious statistical signal.

Among the linkage groups observed in the GWAS were two sets of genes responsible for encoding iron acquisition systems. We observed that genes encoding both the *TonB1*-mediated *Cj0178* (LG2; *Cj0177–Cj0181*) and the *TonB3*-mediated *CfrA* (LG6; *Cj0753c*/*Cj0755*) iron acquisition systems were significantly associated with *C. jejuni* isolates from clinically related CGF subtypes. As is the case in most pathogens, iron acquisition is considered to be a virulence determinant in *C. jejuni* and has been linked to successful colonization *in vivo* (Kim et al., 2003; Palyada et al., 2004; Naikare et al., 2006). *CfrA* has been shown to be capable of transporting a wide variety of structurally different siderophores, which may contribute to the ability of isolates with these genes to colonize a wide variety of hosts/niches (Naikare et al., 2013).

Another linkage group associated with CA and UN subtypes was comprised of genes that encode the pantothenate (vitamin B_5_) biosynthesis pathway and β-lactam antibiotic resistance. LG5 encompasses a total of five genes, including a putative acetyltransferase (*Cj0295*), the *panBCD* operon (*Cj0296c–Cj0298c*), which encodes for the pantothenate (vitamin B_5_) biosynthesis pathway, as well as the gene *bla_OXA-61_* (*Cj0299*), which encodes a protein that confers resistance to β-lactam antibiotics. These genes were recently implicated in host adaptation in *C. jejuni* and *C. coli*, where they were found to be more strongly associated with cattle-specific lineages relative to chicken-specific lineages, possibly as a result of selective pressures created by contemporary and geographically dependent agricultural practices (Sheppard et al., 2013). Although it is generally recognized that chickens are a primary source of human exposure leading to infection, we observed strong statistical signal among CA subtypes for genes previously identified as cattle-associated (Sheppard et al., 2013). Sheppard et al. (2013) suggested that maintenance of these genes in chickens, albeit at a reduced rate, may facilitate rapid-host switching as part of a host-generalist strategy. Moreover, we have observed that a majority of the most prevalent clinically related CGF subtypes, many of which are represented in our GWAS dataset, are associated with both cattle and chickens. This is consistent with the possible role of cattle as an important reservoir for strains that go on to contaminate the chicken production system, ultimately leading to human cases of campylobacteriosis. As this manuscript was being readied for publication, GWAS was used to identify several loci that could be used as “host-segregating” epidemiological markers markers for source attribution (Thépault et al., 2017). Interestingly, one of the loci (*Cj0260c*) was also identified in our analysis. Thus, while our data suggests that presence of this gene is strongly associated with human clinical isolates, data from the study by Thépault et al. further suggests the allelic information appears highly predictive of host source.

### *In Silico* Validation of Putative Diagnostic Marker Genes

**FIGURE 3:**
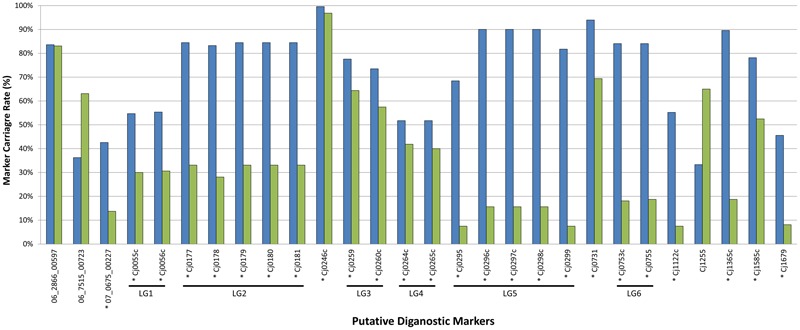
*In silico* validation of putative diagnostic marker genes against expanded CA and NCA cohorts. The putative diagnostic genes identified by GWAS using the original set of 166 genomes were tested for statistical significance with expanded CA (blue bars; *n* = 3,742) and NCA (green bars; *n* = 160) cohorts comprised of additional genomes sequenced in house and from public repositories. Despite the influx of genetically and geographically diverse isolates introduced as part of the expanded dataset, a majority (*n* = 25) of the markers continued to show statistically significant signal with CA subtypes. This suggests that the robust signal detected in the original GWAS analysis stems from genes that appear to have diagnostic value for the identification of *C. jejuni* subtypes with an increased association with campylobacteriosis. ^∗^Denotes genes that showed statistically significant signal with CA subtypes.

Population structure has been identified as a potential confounding factor in GWAS analyses, in that statistically significant associations may ultimately be due to oversampling of certain subpopulations rather than with the phenotypic trait under investigation (Read and Massey, 2014). Since the focus of the current study was the examination of prevalent *C. jejuni* subtypes in Canada in the context of population structure, it was necessary to exclude the possibility that the markers we identified represent a biased distribution resulting from oversampling within certain lineages in the population. The large-scale marker validation that we performed using available WGS data included a dataset comprised of genomes largely from the United Kingdom (3,871/4,280; 90%) and Canada (327/4,280; 8%), and an overwhelming majority of isolates were recovered from human clinical sources (3,559/4,280; 83%), while those from animal (626/4,280; 15%) and environmental (95/4,280; 2%) sources comprised the remainder. A total of 539 CGF subtypes were identified by *in silico* CGF, however, 279 subtypes were novel and had not been previously observed in the C3GFdb and were omitted from the analysis since their epidemiological characteristics could not be determined. Of the remaining 260 CGF subtypes, 38 CGF subtypes (160 genomes) were identified as NCA, nine CGF subtypes (99 genomes) were identified as UN, and 213 CGF subtypes (3,742 isolates) were identified as CA. Despite the influx of genetically and geographically diverse isolates introduced as part of the expanded dataset, a majority (*n* = 25) of the markers in the original GWAS analysis continued to show statistical significance with CA subtypes; on average these markers were present in 73% of CA isolates compared to only 36% of NCA isolates (**Figure 3**). Moreover, results of our combinatorial marker analysis show that as few as four markers could be used in combination to detect up to 90% of CA isolates in the validation dataset, with a modest carriage rate of 21% among NCA isolates. These findings suggest that the robust signal detected in the original GWAS analysis stems from genes that appear to have diagnostic value for the identification of *C. jejuni* subtypes with an increased association with campylobacteriosis.

## Conclusion

A major challenge in the prevention and control of campylobacteriosis is our current inability to identify strains of *C. jejuni* that pose the greatest risk to human health. Addressing this issue would pave the way to better tracking of high-risk strains, leading to a better understanding of their distribution in the food chain and providing critical information towards the development of targeted mitigation strategies to reduce human exposure.

The goal of this study was to identify markers associated with *C. jejuni* lineages known to cause disease in humans and that have a high prevalence in Canada. The genomes of 166 isolates representing 34 highly prevalent *C. jejuni* subtypes were sequenced and a GWAS was performed to identify 28 genes significantly associated with highly prevalent and clinically-related *C. jejuni* subtypes. While some putative gene markers identified as part of this study have previously been associated with important aspects of *C. jejuni* biology including iron acquisition and vitamin B_5_ biosynthesis, others represent putative proteins associated with catalysis and transport, which may play roles in processes important for infection and warrant further investigation.

Although these genes were identified within a dataset of Canadian origin, 25 of them continued to display strong statistical significance when validated against a more genetically and geographically diverse dataset. This suggests that they may represent robust markers for clinically-associated *C. jejuni* subtypes, paving the way for future development of molecular assays for rapid identification of *C. jejuni* strains that pose an increased risk to human health.

## Author Contributions

CB participated in all aspects of laboratory and *in silico* analyses and drafted the manuscript; AW and SM participated in data analysis and drafting of the manuscript; PK, DB, and BH assisted with various aspects of bioinformatics analyses; VG, WA, JT, DI, and ET contributed to study design and writing the manuscript.

## Conflict of Interest Statement

The authors declare that the research was conducted in the absence of any commercial or financial relationships that could be construed as a potential conflict of interest.

## Footnotes

1 https://bitbucket.org/peterk87/genomefisher/wiki/Home
