# Supplementary material for: A Genome-Wide Association Study to Identify Diagnostic Markers for Human Pathogenic Campylobacter jejuni Strains
Source: Front Microbiol. 2017 Jun 30;8:1224. doi: 10.3389/fmicb.2017.01224 (PMC5492696; doi:10.3389/fmicb.2017.01224)
Supplement: Supplementary file 1 [file Table_1.DOCX]

Supplementary Table 1. *C. jejuni* isolates selected for whole genome sequencing.

| Strain | CGF Subtype | Clinical Relevance | Host | Isolation Year | Isolation Location | Accession Number |
| --- | --- | --- | --- | --- | --- | --- |
| C3GFDB009514 | 0083.007.001 | CA | Human | 2007 | Ontario | SAMN06270746 |
| C3GFDB009574 | 0083.007.001 | CA | Chicken | 2007 | Ontario | SAMN06270747 |
| C3GFDB008268 | 0103.001.002 | CA | Cow | 2010 | Ontario | SAMN06270748 |
| C3GFDB016493 | 0253.001.002 | CA | Human | 2005 | Alberta | SAMN06270749 |
| C3GFDB016524 | 0077.001.003 | CA | Human | 2005 | Alberta | SAMN06270750 |
| C3GFDB016539 | 0077.001.003 | CA | Human | 2005 | Alberta | SAMN06270751 |
| C3GFDB016546 | 0253.001.002 | CA | Human | 2005 | Alberta | SAMN06270752 |
| C3GFDB016564 | 0933.004.002 | CA | Human | 2005 | Alberta | SAMN06270753 |
| C3GFDB016571 | 0044.003.001 | CA | Human | 2005 | Alberta | SAMN06270754 |
| C3GFDB016572 | 0169.001.002 | CA | Human | 2005 | Alberta | SAMN06270755 |
| C3GFDB016592 | 0169.001.002 | CA | Human | 2005 | Alberta | SAMN06270756 |
| C3GFDB016601 | 0169.001.002 | CA | Human | 2005 | Alberta | SAMN06270757 |
| C3GFDB016634 | 0169.001.002 | CA | Human | 2005 | Alberta | SAMN06270758 |
| C3GFDB017536 | 0269.004.001 | CA | Human | 2004 | Alberta | SAMN06270759 |
| C3GFDB017537 | 0269.004.001 | CA | Human | 2004 | Alberta | SAMN06270760 |
| C3GFDB017540 | 0044.003.001 | CA | Human | 2004 | Alberta | SAMN06270761 |
| C3GFDB017542 | 0173.004.001 | CA | Human | 2004 | Alberta | SAMN06270762 |
| C3GFDB017610 | 0269.004.001 | CA | Human | 2005 | Alberta | SAMN06270763 |
| C3GFDB017620 | 0044.003.001 | CA | Human | 2005 | Alberta | SAMN06270764 |
| C3GFDB017638 | 0169.001.002 | CA | Human | 2005 | Alberta | SAMN06270765 |
| C3GFDB017646 | 0933.004.002 | CA | Human | 2005 | Alberta | SAMN06270766 |
| C3GFDB014399 | 0173.004.001 | CA | Cow | 2004 | Alberta | SAMN06270767 |
| C3GFDB014501 | 0044.003.001 | CA | Cow | 2004 | Alberta | SAMN06270768 |
| C3GFDB014638 | 0269.004.001 | CA | Cow | 2004 | Alberta | SAMN06270769 |
| C3GFDB014681 | 0269.004.001 | CA | Cow | 2005 | Alberta | SAMN06270770 |
| C3GFDB014710 | 0044.003.001 | CA | Cow | 2005 | Alberta | SAMN06270771 |
| C3GFDB014716 | 0169.001.002 | CA | Cow | 2005 | Alberta | SAMN06270772 |
| C3GFDB014747 | 0933.004.002 | CA | Sheep | 2005 | Alberta | SAMN06270773 |
| C3GFDB014751 | 0044.003.001 | CA | Cow | 2005 | Alberta | SAMN06270774 |
| C3GFDB014892 | 0169.001.002 | CA | Chicken | 2006 | Alberta | SAMN06270775 |
| C3GFDB014897 | 0169.001.002 | CA | Chicken | 2006 | Alberta | SAMN06270776 |
| C3GFDB014986 | 0269.004.001 | CA | Cow | 2011 | Alberta | SAMN06270777 |
| C3GFDB015015 | 0269.004.001 | CA | Cow | 2011 | Alberta | SAMN06270778 |
| C3GFDB015022 | 0253.001.002 | CA | Cow | 2011 | Alberta | SAMN06270779 |
| C3GFDB015057 | 0253.001.002 | CA | Cow | 2011 | Alberta | SAMN06270780 |
| C3GFDB015090 | 0253.001.002 | CA | Cow | 2011 | Alberta | SAMN06270781 |
| C3GFDB015145 | 0269.004.001 | CA | Cow | 2011 | Alberta | SAMN06270782 |
| C3GFDB015154 | 0269.004.001 | CA | Cow | 2011 | Alberta | SAMN06270783 |
| C3GFDB015165 | 0269.004.001 | CA | Cow | 2011 | Alberta | SAMN06270784 |
| C3GFDB015192 | 0269.004.001 | CA | Cow | 2011 | Alberta | SAMN06270785 |
| C3GFDB015200 | 0269.004.001 | CA | Cow | 2011 | Alberta | SAMN06270786 |
| C3GFDB015228 | 0169.001.002 | CA | Cow | 2012 | Alberta | SAMN06270787 |
| C3GFDB015257 | 0169.001.002 | CA | Cow | 2012 | Alberta | SAMN06270788 |
| C3GFDB015303 | 0169.001.002 | CA | Cow | 2012 | Alberta | SAMN06270789 |
| C3GFDB015390 | 0173.004.001 | CA | Cow | 2012 | Alberta | SAMN06270790 |
| C3GFDB016536 | 0982.001.002 | CA | Human | 2005 | Alberta | SAMN06270791 |
| C3GFDB016537 | 0982.001.002 | CA | Human | 2005 | Alberta | SAMN06270792 |
| C3GFDB014656 | 0982.001.002 | CA | Cow | 2005 | Alberta | SAMN06270793 |
| C3GFDB014661 | 0982.001.002 | CA | Cow | 2005 | Alberta | SAMN06270794 |
| C3GFDB015708 | 0982.001.002 | CA | Water | 2005 | Alberta | SAMN06270795 |
| C3GFDB016010 | 0982.001.002 | CA | Water | 2010 | Alberta | SAMN06270796 |
| C3GFDB014551 | 0817.003.001 | NCA | Goose | 2004 | Alberta | SAMN06270797 |
| C3GFDB014554 | 0817.003.001 | NCA | Goose | 2004 | Alberta | SAMN06270798 |
| C3GFDB014559 | 0817.003.001 | NCA | Goose | 2004 | Alberta | SAMN06270799 |
| C3GFDB015571 | 0811.009.002 | NCA | Water | 2004 | Alberta | SAMN06270800 |
| C3GFDB015649 | 0817.003.001 | NCA | Water | 2004 | Alberta | SAMN06270801 |
| C3GFDB015666 | 0811.009.002 | NCA | Water | 2004 | Alberta | SAMN06270802 |
| C3GFDB015668 | 0817.003.001 | NCA | Water | 2004 | Alberta | SAMN06270803 |
| C3GFDB014630 | 0811.009.002 | NCA | Goose | 2004 | Alberta | SAMN06270804 |
| C3GFDB016172 | 0811.009.002 | NCA | Water | 2006 | British Columbia | SAMN06270805 |
| C3GFDB015754 | 0811.009.002 | NCA | Water | 2006 | Alberta | SAMN06270806 |
| C3GFDB016266 | 0811.009.002 | NCA | Water | 2006 | British Columbia | SAMN06270807 |
| C3GFDB014923 | 0811.008.001 | NCA | Goose | 2006 | Alberta | SAMN06270808 |
| C3GFDB014927 | 0811.008.001 | NCA | Duck | 2006 | Alberta | SAMN06270809 |
| C3GFDB015823 | 0811.008.001 | NCA | Water | 2006 | Alberta | SAMN06270810 |
| C3GFDB019796 | 0844.001.001 | NCA | Water | 2006 | Ontario | SAMN06270811 |
| C3GFDB019832 | 0811.009.002 | NCA | Water | 2006 | Ontario | SAMN06270812 |
| C3GFDB015843 | 0817.003.001 | NCA | Water | 2006 | Alberta | SAMN06270813 |
| C3GFDB015846 | 0811.009.002 | NCA | Water | 2006 | Alberta | SAMN06270814 |
| C3GFDB016311 | 0844.001.001 | NCA | Water | 2006 | British Columbia | SAMN06270815 |
| C3GFDB019847 | 0811.009.002 | NCA | Water | 2006 | Ontario | SAMN06270816 |
| C3GFDB015878 | 0817.003.001 | NCA | Water | 2006 | Alberta | SAMN06270817 |
| C3GFDB016325 | 0811.009.002 | NCA | Water | 2006 | British Columbia | SAMN06270818 |
| C3GFDB015958 | 0811.008.001 | NCA | Water | 2007 | Alberta | SAMN06270819 |
| C3GFDB016401 | 0844.001.001 | NCA | Water | 2007 | British Columbia | SAMN06270820 |
| C3GFDB016403 | 0844.001.001 | NCA | Water | 2007 | British Columbia | SAMN06270821 |
| C3GFDB015968 | 0844.001.001 | NCA | Water | 2007 | Alberta | SAMN06270822 |
| C3GFDB015981 | 0811.008.001 | NCA | Water | 2007 | Alberta | SAMN06270823 |
| C3GFDB015988 | 0817.003.001 | NCA | Water | 2007 | Alberta | SAMN06270824 |
| C3GFDB016437 | 0817.003.001 | NCA | Water | 2007 | British Columbia | SAMN06270825 |
| C3GFDB019988 | 0811.008.001 | NCA | Water | 2008 | Ontario | SAMN06270826 |
| C3GFDB020240 | 0811.008.001 | NCA | Water | 2009 | Ontario | SAMN06270827 |
| C3GFDB016025 | 0811.009.002 | NCA | Water | 2010 | Alberta | SAMN06270828 |
| C3GFDB020625 | 0811.009.002 | NCA | Water | 2010 | Ontario | SAMN06270829 |
| C3GFDB021309 | 0811.009.002 | NCA | Water | 2010 | Quebec | SAMN06270830 |
| C3GFDB020827 | 0811.008.001 | NCA | Water | 2011 | Ontario | SAMN06270831 |
| C3GFDB009352 | 0926.002.001 | UN | Chicken | 2005 | Ontario | SAMN06270832 |
| C3GFDB009426 | 0926.002.001 | UN | Human | 2006 | Ontario | SAMN06270833 |
| C3GFDB009442 | 0173.002.004 | UN | Human | 2006 | Ontario | SAMN06270834 |
| C3GFDB009472 | 0735.005.001 | UN | Human | 2006 | Ontario | SAMN06270835 |
| C3GFDB009494 | 0853.011.001 | UN | Human | 2007 | Ontario | SAMN06270836 |
| C3GFDB008237 | 0960.003.002 | UN | Cow | 2010 | Ontario | SAMN06270837 |
| C3GFDB008715 | 0893.001.001 | UN | Chicken | 2011 | Ontario | SAMN06270838 |
| C3GFDB008759 | 0173.002.004 | UN | Chicken | 2011 | Ontario | SAMN06270839 |
| C3GFDB008776 | 0923.002.001 | UN | Chicken | 2011 | Ontario | SAMN06270840 |
| C3GFDB008798 | 0882.005.001 | UN | Turkey | 2011 | Ontario | SAMN06270841 |
| C3GFDB008833 | 0882.005.001 | UN | Chicken | 2011 | British Columbia | SAMN06270842 |
| C3GFDB008868 | 0893.001.001 | UN | Chicken | 2011 | British Columbia | SAMN06270843 |
| C3GFDB016476 | 0695.006.001 | UN | Human | 2005 | Alberta | SAMN06270844 |
| C3GFDB016480 | 0253.004.001 | UN | Human | 2005 | Alberta | SAMN06270845 |
| C3GFDB016487 | 0695.006.001 | UN | Human | 2005 | Alberta | SAMN06270846 |
| C3GFDB016492 | 0933.008.001 | UN | Human | 2005 | Alberta | SAMN06270847 |
| C3GFDB016521 | 0695.006.001 | UN | Human | 2005 | Alberta | SAMN06270848 |
| C3GFDB016545 | 0083.001.002 | UN | Human | 2005 | Alberta | SAMN06270849 |
| C3GFDB016553 | 0083.001.002 | UN | Human | 2005 | Alberta | SAMN06270850 |
| C3GFDB016561 | 0083.001.002 | UN | Human | 2005 | Alberta | SAMN06270851 |
| C3GFDB016566 | 0960.007.001 | UN | Human | 2005 | Alberta | SAMN06270852 |
| C3GFDB016580 | 0926.002.001 | UN | Human | 2005 | Alberta | SAMN06270853 |
| C3GFDB016583 | 0253.004.001 | UN | Human | 2005 | Alberta | SAMN06270854 |
| C3GFDB016602 | 0933.008.001 | UN | Human | 2005 | Alberta | SAMN06270855 |
| C3GFDB016630 | 0083.001.002 | UN | Human | 2005 | Alberta | SAMN06270856 |
| C3GFDB017566 | 0695.006.001 | UN | Human | 2004 | Alberta | SAMN06270857 |
| C3GFDB017618 | 0260.007.001 | UN | Human | 2005 | Alberta | SAMN06270858 |
| C3GFDB015479 | 0731.001.005 | UN | Water | 2004 | Alberta | SAMN06270859 |
| C3GFDB014430 | 0949.001.002 | UN | Cow | 2004 | Alberta | SAMN06270860 |
| C3GFDB014444 | 0949.001.002 | UN | Cow | 2004 | Alberta | SAMN06270861 |
| C3GFDB015494 | 0695.006.001 | UN | Water | 2004 | Alberta | SAMN06270862 |
| C3GFDB014498 | 0960.007.001 | UN | Cow | 2004 | Alberta | SAMN06270863 |
| C3GFDB014510 | 0695.006.001 | UN | Cow | 2004 | Alberta | SAMN06270864 |
| C3GFDB014519 | 0238.007.002 | UN | Sheep | 2004 | Alberta | SAMN06270865 |
| C3GFDB015508 | 0949.001.002 | UN | Sewage | 2004 | Alberta | SAMN06270866 |
| C3GFDB014531 | 0731.001.005 | UN | Cat | 2004 | Alberta | SAMN06270867 |
| C3GFDB015612 | 0904.002.002 | UN | Water | 2004 | Alberta | SAMN06270868 |
| C3GFDB015628 | 0933.008.001 | UN | Sewage | 2004 | Alberta | SAMN06270869 |
| C3GFDB014616 | 0083.001.002 | UN | Chicken | 2004 | Alberta | SAMN06270870 |
| C3GFDB014624 | 0083.001.002 | UN | Chicken | 2004 | Alberta | SAMN06270871 |
| C3GFDB014647 | 0731.001.005 | UN | Cow | 2005 | Alberta | SAMN06270872 |
| C3GFDB014687 | 0695.006.001 | UN | Cow | 2005 | Alberta | SAMN06270873 |
| C3GFDB014743 | 0957.001.001 | UN | Sheep | 2005 | Alberta | SAMN06270874 |
| C3GFDB014764 | 0957.001.001 | UN | Cow | 2005 | Alberta | SAMN06270875 |
| C3GFDB014772 | 0957.001.001 | UN | Sheep | 2005 | Alberta | SAMN06270876 |
| C3GFDB018263 | 0535.001.003 | UN | Water | 2006 | New Brunswick | SAMN06270877 |
| C3GFDB019677 | 0535.001.003 | UN | Water | 2006 | Ontario | SAMN06270878 |
| C3GFDB014863 | 0695.006.001 | UN | Cow | 2006 | Alberta | SAMN06270879 |
| C3GFDB015779 | 0960.007.001 | UN | Water | 2006 | Alberta | SAMN06270880 |
| C3GFDB014881 | 0926.002.001 | UN | Goose | 2006 | Alberta | SAMN06270881 |
| C3GFDB014887 | 0926.002.001 | UN | Dog | 2006 | Alberta | SAMN06270882 |
| C3GFDB014933 | 0926.002.001 | UN | Duck | 2006 | Alberta | SAMN06270883 |
| C3GFDB014939 | 0926.002.001 | UN | Chicken | 2006 | Alberta | SAMN06270884 |
| C3GFDB018269 | 0535.001.003 | UN | Water | 2006 | New Brunswick | SAMN06270885 |
| C3GFDB019812 | 0957.001.001 | UN | Water | 2006 | Ontario | SAMN06270886 |
| C3GFDB015839 | 0904.002.002 | UN | Water | 2006 | Alberta | SAMN06270887 |
| C3GFDB019857 | 0957.001.001 | UN | Water | 2006 | Ontario | SAMN06270888 |
| C3GFDB015915 | 0960.007.001 | UN | Water | 2007 | Alberta | SAMN06270889 |
| C3GFDB019903 | 0735.005.001 | UN | Water | 2007 | Ontario | SAMN06270890 |
| C3GFDB014971 | 0949.001.002 | UN | Cow | 2009 | Alberta | SAMN06270891 |
| C3GFDB014974 | 0949.001.002 | UN | Cow | 2009 | Alberta | SAMN06270892 |
| C3GFDB020075 | 0731.001.005 | UN | Water | 2009 | Ontario | SAMN06270893 |
| C3GFDB020274 | 0957.001.001 | UN | Water | 2009 | Ontario | SAMN06270894 |
| C3GFDB020424 | 0535.001.003 | UN | Water | 2010 | Ontario | SAMN06270895 |
| C3GFDB021276 | 0535.001.003 | UN | Water | 2010 | Quebec | SAMN06270896 |
| C3GFDB020474 | 0957.001.001 | UN | Water | 2010 | Ontario | SAMN06270897 |
| C3GFDB016023 | 0535.001.003 | UN | Water | 2010 | Alberta | SAMN06270898 |
| C3GFDB020618 | 0957.001.001 | UN | Water | 2010 | Ontario | SAMN06270899 |
| C3GFDB015045 | 0695.006.001 | UN | Cow | 2011 | Alberta | SAMN06270900 |
| C3GFDB015120 | 0695.006.001 | UN | Cow | 2011 | Alberta | SAMN06270901 |
| C3GFDB020782 | 0957.001.001 | UN | Water | 2011 | Ontario | SAMN06270902 |
| C3GFDB018644 | 0904.002.002 | UN | Raccoon | 2011 | Ontario | SAMN06270903 |
| C3GFDB018673 | 0904.002.002 | UN | Raccoon | 2011 | Ontario | SAMN06270904 |
| C3GFDB018718 | 0904.002.002 | UN | Raccoon | 2011 | Ontario | SAMN06270905 |
| C3GFDB018774 | 0535.001.003 | UN | Raccoon | 2011 | Ontario | SAMN06270906 |
| C3GFDB020936 | 0957.001.001 | UN | Water | 2011 | Ontario | SAMN06270907 |
| C3GFDB015346 | 0695.006.001 | UN | Cow | 2012 | Alberta | SAMN06270908 |
| C3GFDB015373 | 0695.006.001 | UN | Cow | 2012 | Alberta | SAMN06270909 |
| C3GFDB015427 | 0695.006.001 | UN | Horse | 2012 | Alberta | SAMN06270910 |
| C3GFDB015431 | 0695.006.001 | UN | Horse | 2012 | Alberta | SAMN06270911 |
