# Supplementary material for: A Genome-Wide Association Study to Identify Diagnostic Markers for Human Pathogenic Campylobacter jejuni Strains
Source: Front Microbiol. 2017 Jun 30;8:1224. doi: 10.3389/fmicb.2017.01224 (PMC5492696; doi:10.3389/fmicb.2017.01224)
Supplement: Supplementary file 2 [file Table_2.DOCX]

Supplementary Table 2. Reference completed and manually-annotated *C. jejuni* genomes used for pangenome construction.

| Accession Number | Species | Strain | Reference |
| --- | --- | --- | --- |
| AL111168.1 | *C. jejuni* jejuni | NCTC 11168 | Parkhill et al., 2000 |
| NC_003912.7 | *C. jejuni* jejuni | RM1221 | Fouts et al., 2005 |
| NC_008787.1 | *C. jejuni* jejuni | 81-176 | Direct Submission |
| NC_009707.1 | *C. jejuni* doylei | 269.97 | Direct Submission |
| NC_009839.1 | *C. jejuni* jejuni | 81116 | Direct Submission |

**References**

Fouts, D.E., Mongodin, E.F., Mandrell, R.E., Miller, W.G., Rasko, D.A., Ravel, J., Brinkac, L.M., DeBoy, R.T., Parker, C.T., Daugherty, S.C., et al. (2005). Major structural differences and novel potential virulence mechanisms from the genomes of multiple *Campylobacter* species. PLoS Biol *3*, e15.

Parkhill, J., Wren, B.W., Mungall, K., Ketley, J.M., Churcher, C., Basham, D., Chillingworth, T., Davies, R.M., Feltwell, T., Holroyd, S., et al. (2000). The genome sequence of the food-borne pathogen *Campylobacter jejuni* reveals hypervariable sequences. Nature *403*, 665–668.
